# Preserving the pulmonary valve in Tetralogy of Fallot repair: Reconsidering the indication for valve‐sparing

**DOI:** 10.1111/jocs.17156

**Published:** 2022-11-15

**Authors:** Umar Siddiqi, Adedotun Adewale, Emily Pena, Kelci Schulz, Michel Ilbawi, Chawki El‐Zein, Luca Vricella, Narutoshi Hibino

**Affiliations:** ^1^ Comer Children's Hospital University of Chicago Chicago Illinois USA; ^2^ Advocate Children's Hospital Advocate Children's Heart Institute Oak Lawn Illinois USA

**Keywords:** pulmonary valve, Tetralogy of Fallot, transannular patch, valve‐sparing

## Abstract

**Background:**

Tetralogy of Fallot (TOF) repair is a frequent procedure, and although valve‐sparing (VS) repair is preferred, determining which patients can successfully undergo this operation remains controversial. We sought to identify parameters to determine a selective, accurate indication for VS repair.

**Methods:**

We reviewed 71 patients (82%) undergoing VS repair. We analyzed hemodynamic data, intraoperative reports, and follow‐up echocardiography results to identify acceptable indications. Patients requiring pulmonary valve (PV) reintervention versus no reintervention were compared.

**Results:**

PV annulus size at repair was *z*‐score of −2.0 (−5.3, 1.3). Approximately half (51%) had a *z*‐score less than −2. Cox regression results showed this was not a risk factor for reintervention (*p* = .59). Overall, 1‐, 3‐, 5‐, and 10‐year freedom from PV reintervention rates were 95.8%, 92.8%, 91% and 77.8%, respectively. Residual pulmonary stenosis (PS) at initial repair was relatively higher in the reintervention group compared with no reintervention group (40 [28, 51] mmHg vs. 30 [22, 37] mmHg; *p* = .08). For patients with residual PS, pressure gradient (PG) was consistent over time across both groups (PV reintervention: −3 [−15, 8] mmHg vs. no reintervention: 0 [−9, 8] mmHg). The risk of PV reintervention is 3.7‐fold higher when the PG from intraoperative TEE is greater than 45 mmHg (*p* = .04).

**Conclusions:**

Our review of the midterm outcomes of expanded indication for VS suggests intraoperative decision to convert to transannular patch is warranted if intraoperative postprocedure TEE PG is greater than 45 mmHg or RV pressure is higher than half of systemic pressure to prevent reintervention.

## 1 INTRODUCTION

Tetralogy of Fallot (TOF) is the most common cyanotic heart disease with an estimated incidence of about 1660 babies per year in the United States, approximately 0.19 to 0.26 in 1000 livebirths.^1^ The optimal surgical approach for complete repair of TOF is valve‐sparing (VS) repair, but the criteria for selecting which patients can undergo VS and which will require transannular patch (TAP) reconstruction are controversial. The spectrum of residual hemodynamic lesions ultimately depends on the initial surgical approach utilized for the complete primary repair, with the goal being the absence of both pulmonary stenosis (PS) and pulmonary regurgitation (PR). Many studies have sought to identify key factors that influence these surgical techniques.^2^, ^3^, ^4^

Historically, TAP reconstruction of the right ventricular outflow tract (RVOT) was more frequently performed.^5^ This procedure was thought to be more benign at the time, but the development of chronic right ventricular volume overloading from progressive pulmonary insufficiency is now a well‐established complication of this technique.^6^ Often late sequelae encountered with this surgical approach include right ventricle (RV) dilation, RV diastolic dysfunction, RV fibrosis, tricuspid regurgitation, arrhythmias, and RV ventricular dysfunction.^7^ More recently there has been a tendency towards preservation of the pulmonary valve (PV) function to protect RV function.^8^, ^9^ Recent studies, such as that by Bacha et al., showed that the PVS approach in well‐selected patients significantly reduced the incidence of moderate to severe PR and other related complications of TAP repair and would have acceptable PS.^10^, ^11^, ^12^, ^13^

However, there are unanswered questions of how much residual PS is too much to leave behind, and how much PR is acceptable when the indication of VS procedure is stretched.^14^ Moderate to severe PS is poorly tolerated in infants and children and needs relatively early interventions to relieve this obstruction at PV level.^15^ TOF surgical strategy has improved over the years due to an acute cognizance of the harmful effects of large RV incisions, including myocardial and coronary injury, RV dysfunction, and arrhythmias.^16^ Moreover, MRI has been used as an earlier indication for pulmonary valve reintervention (PVR).^17^, ^18^ These historical changes in the management of TOF could affect the indication of pulmonary VS repair. Parameters to decide whether to opt for VS repair or a TAP repair still largely remain to be determined and so the predictors of future PV intervention in these patients. Expanded indications are less selective, leading to a higher rate of VS procedures for a broader variety of patients. The goal of this study is to reconsider the indication of PVS by reviewing the midterm outcomes of our expanded indication of VS procedure for complete TOF repair.

## 2 METHODS

This single‐center, retrospective study was approved by Advocate Christ Hospital internal review board. The need for written informed consent was waived for the retrospective nature of this study.

### 2.1 Surgical technique

Overall, there have been no significant changes in our surgical technique in complete TOF repair in recent years. We attempt VS repair whenever possible with resection of septal and parietal muscle bands obstructing the RVOT. This often also involves careful inspection of the tricuspid valve, pulmonary valvotomy and commissurotomy, main pulmonary artery patch plasty for supravalvular stenosis and reevaluation of the PV annulus diameter using the Hegar dilator. Further resection of abnormal muscle band at RVOT is performed as much as possible through a separate RV incision extended close to annulus if needed. Finally, decision on what approach for complete repair is often taken intraoperatively based on PV annulus after dilation with Hegar dilator. The morphology of the PV is also evaluated intraoperatively. Mild to moderate residual stenosis or regurgitation by transesophageal echocardiogram (TEE) and RV pressure of less than 75% of systemic pressure were accepted at the end of the procedure. Two cases with dysplastic PV and one case of systemic RV pressure due to small annulus after PV repair were converted to transannular patch. For one patient, part of the annulus was divided to gain good visualization of the subpulmonary area, and this was reapproximated with two interrupted 6‐0 Prolene sutures.

If patients have an RVOT stent or an extremely small annulus, the surgeon will proceed straight to TA patch. A patient may be returned to bypass and switched to TA patch if right ventricular pressure (RVP) is greater than 75% of systemic pressure after coming off pump. If the annulus size is less than 6 mm, we convert it to TA patch. Original leaflet structure was not a relevant component of our decision‐making process. We did not use balloon valvuloplasty during surgery.

In cases with residual subvalvular obstruction despite maximal muscle resection, the superior edge of the RV incision extends the annulus as much as possible. A T‐shaped incision parallel to the annulus is added to remove subvalvular obstruction for patients with small PV annulus.

### 2.2 Patients

All patients with TOF who underwent pulmonary VS repair at the Advocate Children's Heart Institute, Oak Lawn between February 2009 and November 2018 were identified from the cardiac surgical electronic database. Patients for whom adequate echocardiographic and clinical data follow‐up were available were included in the study.

The data collection was performed between February 2021 and May 2021. Exclusion criteria involved patients with TOF with pulmonary atresia, absent PV, or placement of right ventricle to pulmonary artery conduit in case of large coronary artery branch crossing RVOT or unavailability of adequate follow‐up data.

### 2.3 Endpoints

Primary outcome of interest was identified as surgical, or catheter‐based PV intervention or mortality. Secondary outcome of interest included the development of moderate to severe PV stenosis or insufficiency.

### 2.4 Statistical analysis

A retrospective chart review of 71 patients who underwent VS TOF repair (82% of all TOF repairs) was performed. We analyzed the hemodynamic data, intraoperative reports, and follow‐up echocardiography results to identify the acceptable indication for a VS procedure. A comparison of patients requiring PV reintervention (surgical or transcatheter) versus no reintervention was performed. Numeric data were compared using means and standard deviations and categorical data using frequency/percentages. Statistical analyses were performed with IBM SPSS Statistics v. 26.0 (IBM, Armonk, NY). Two‐sided *p* values <.05 were considered statistically significant. Continuous variables were compared using Mann–Whitney *U* test and expressed as median and interquartile range. Categorical variables were expressed as numbers and percentages and compared with Fisher's exact test. Survival times were compared using log‐rank tests and presented in Kaplan–Meier curves. Using Cox's proportional hazard model, we compared the rates of PV intervention between both groups when TEE PG > 45.

### 2.5 *Z*‐score calculation

The preoperative pulmonary annulus *z*‐score for all study patients was determined using the online calculator based on the Detroit score.^19^

## 3 RESULTS

### 3.1 Patient characteristics

Baseline characteristics are shown in Table 1. The mean age of VS patients at TOF repair was 4 (0.75, 18) months old with a mean PV size *z*‐score of −2.0 (−5.3, 1.3). After commissurotomy, the mean PV size *z*‐score was −0.93 (−3.25, 1.35). Nearly 78% of our study population had a bicuspid PV at the time of repair. The median post‐PV commissurotomy annulus size was 8 mm, and less than half (44%) of the patients required additional PV intervention. Almost half of our patients (47%) had a *z*‐score less than −2 and RVP less than 50% of systemic pressure.

**Table 1:** Baseline characteristics at index surgery

Characteristic	Valve sparing (*N* = 71)
Age, months	4 (0.75, 18)
Weight, kg	5.9 (3.1, 9.6)
Height, cm	59 (47, 79)
PV annulus size, mm	7.2 (4.4, 12)
*Z*‐score	−2 (−5.3, 1.3)
Post PV commissurotomy annulus size, mm	8 (5.5, 12)
Valve cusps	2.2 (2, 3)
Pre‐ or peri‐operative palliative surgery	
Yes	7 (10%)
No	63 (90%)
Additional pulmonary valve procedure	
Commissurotomy, dilation with Hegar	31 (44%)
None	39 (56%)

*Note*: Continuous variables expressed as mean (range).

Abbreviation: PV, pulmonary valve.

For one patient, it was believed that the valve could be spared, so a VS procedure was attempted. However, by the end of the procedure, the pressure in the right ventricle was systemic. Echocardiogram further confirmed the presence of severe hypoplasia at the annulus as well as the aforementioned gradient. Therefore, it was decided to return to bypass and make a transannular incision. This patient was not included in the present analysis as they were converted to TA patch. In total, six patients (2, dysplastic valve + 4, small annulus) converted to TAP after trying VS.

### 3.2 Freedom from reintervention and hemodynamics

**Figure 1:**
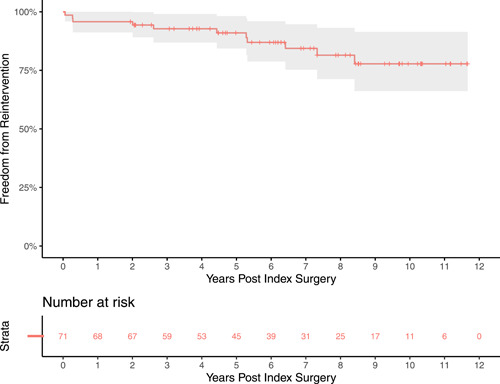
Freedom from pulmonary valve reintervention. Freedom from reintervention was favorable. Ten‐year freedom was 77.8% (95% CI 66.1–91.4). Median follow‐up time was 6.28 years.

Freedom from reintervention for PV including catheter procedure and surgical valve replacement is displayed in Figure 1. Estimates of freedom from PVR at 1, 3, 5, and 10 years were 95.8% (95% confidence interval [CI] 91.2–100), 92.8%, (95% CI 86.9–99.1), 91% (95% CI 84.3–98.2), and 77.8% (95% CI 66.1–91.4), respectively. The median time of follow‐up was 6.28 years.

**Figure 2:**
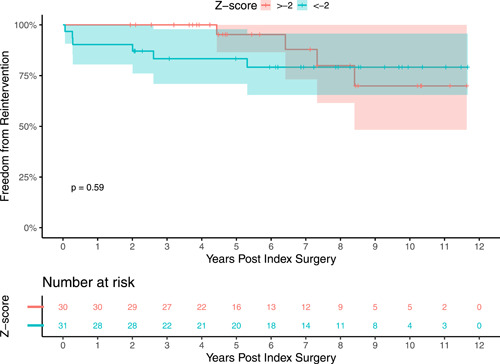
Freedom from reintervention by *z*‐score. Patients were divided into (1) *z*‐score > −2 or (2) *z*‐score < −2. Freedom from pulmonary valve reintervention was statistically comparable between the two groups.

Approximately half of the patients (51%) had a *z*‐score less than −2. Cox regression results using this score as a cutoff found comparable freedom from reintervention in the two groups: HR 1.41 (95% CI 0.4–5), *p* = .59 (Figure 2).

**Figure 3:**
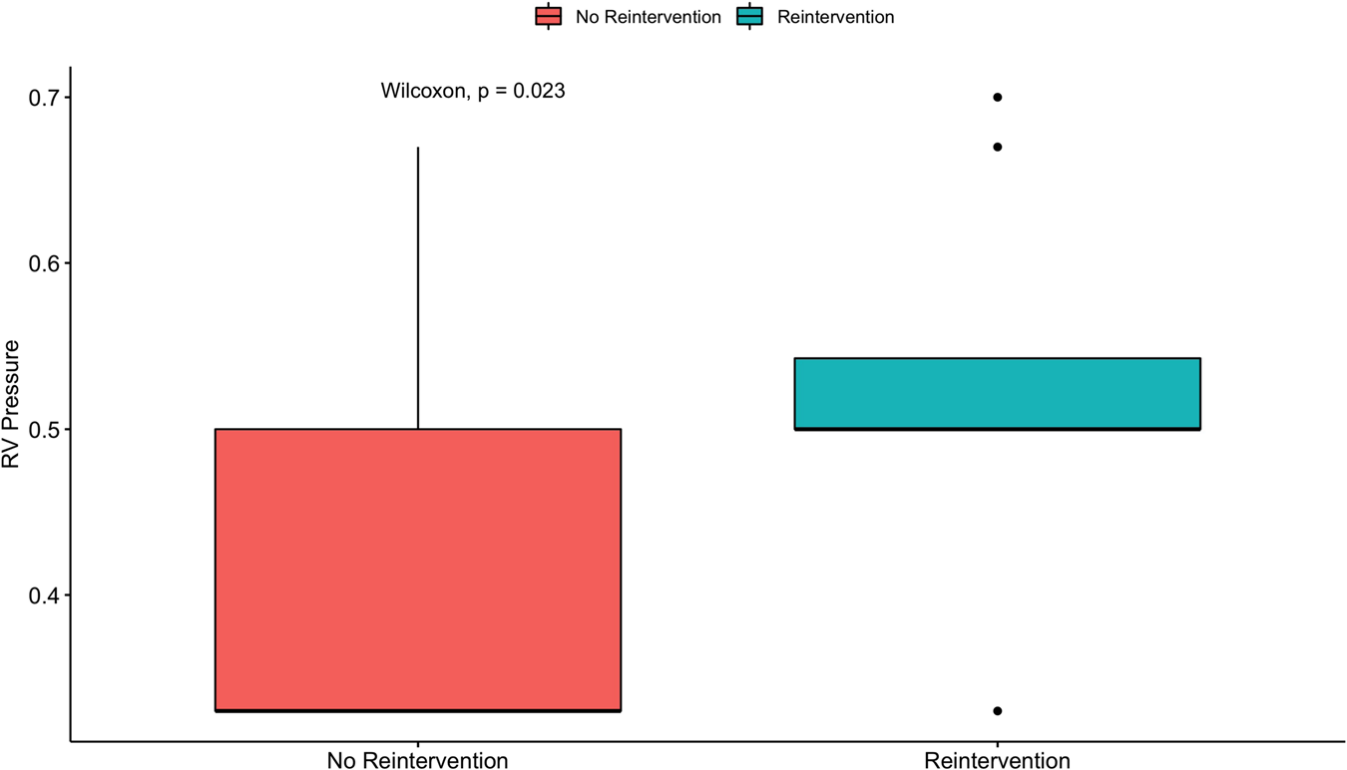
Postoperative right ventricular pressure (RVP) in pulmonary valve re‐intervention group and no reintervention group. RVP after the operation was significantly lower in patients who did not require reintervention compared to those who did. RV, right ventricle.

Residual PS at the time of initial TOF repair, as measured by RVOT flow acceleration echocardiography on intraoperative TEE, was relatively higher in the reintervention group compared with no‐reintervention group (40 [28, 51] mmHg vs. 30 [22, 37] mmHg; *p* = .088). Likewise, patients requiring reintervention had significantly higher direct pressure measurement of the ratio for RV/Ao at the initial TOF repair than those who did not (0.5 [0.5, 0.54] vs. 0.33 [0.33, 0.5], *p* = .02), which is shown in Figure 3.

**Figure 4:**
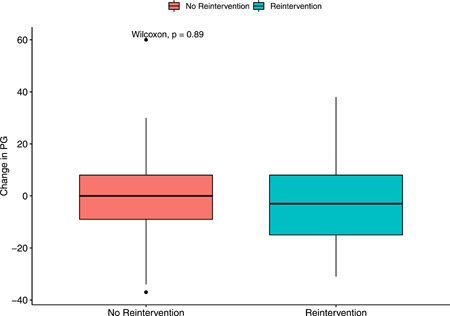
Change in pressure gradient (PG) between initial postoperative ECHO and outpatient follow‐up ECHO. The change in PG was statistically comparable for patients who required reintervention as well as those who did not. ECHO, echocardiogram.

**Figure 5:**
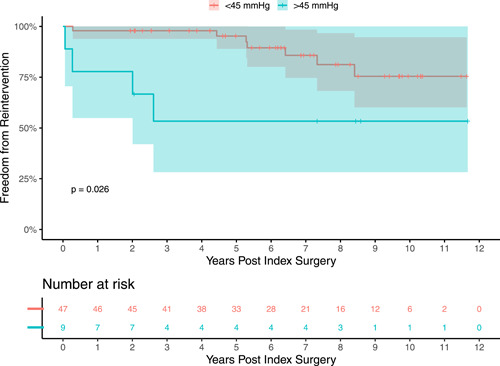
Freedom from reintervention by residual stenosis. Patients were divided into (1) residual gradient <45 or (2) residual gradient >45. Freedom from pulmonary valve reintervention was significantly lower in the group with residual gradient >45.

There was limited change of the PV pressure gradient (PG) over time across both groups (PV reintervention: −3 [−15, 8] mmHg vs. no‐reintervention: 0 [−9, 8] mmHg; see Figure 4). Using a cox regression model, the risk of PV reintervention was 3.7‐fold (95% CI 1.1–13) higher when the PG from intraoperative echo is greater than 45 mmHg (*p* = .04; Figure 5). Most patients (61.7%) had none to mild regurgitation at follow‐up.

**Figure 6:**
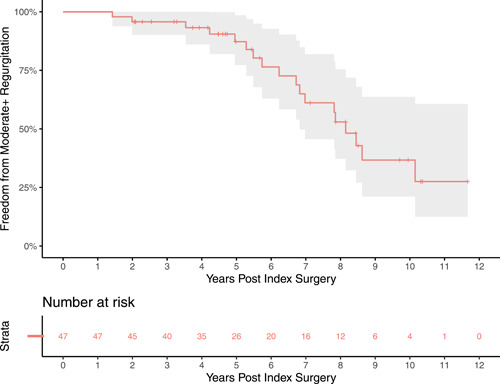
Freedom from moderate + regurgitation. The endpoint was defined as either moderate or severe pulmonary regurgitation. Freedom from greater than moderate regurgitation was much lower than that of reintervention.

**Figure 7:**
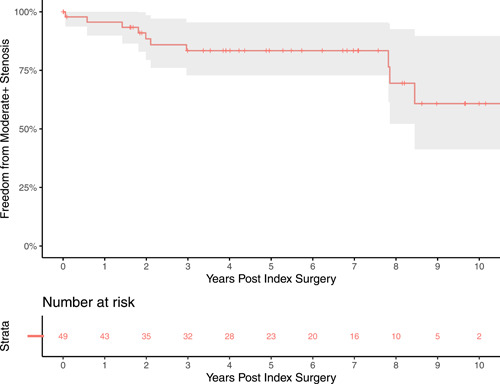
Freedom from moderate + stenosis. The endpoint was defined as either moderate or severe pulmonary stenosis. Freedom from greater than moderate stenosis was acceptable.

Freedom from more than moderate pulmonary regurgitation is displayed in Figure 6. The median follow‐up time for echocardiography was 5.44 years. The median time to event for patients who experienced the endpoint was 6.48 years. Freedom from more than moderate PS is shown in Figure 7. The median time to event for patients who experienced the endpoint was 2.05 years.

## 4 DISCUSSION

Overall survival after complete repair of TOF has significantly improved over the last few decades (>90% 20‐year survival), with improvement of management especially preserving right ventricle function.^20^ A recent study by Smith et al. found excellent survival in patients undergoing simple TOF procedure, including 94.5% survival at 25‐year post‐surgery.^21^ This is a dramatic improvement relative to the 77% 30‐year survival reported in 1986.^22^

One of the significant advancements in management over time has been VS procedure to prevent PV insufficiency. Progressive PV insufficiency caused by loss of integrity of PV annulus has been shown to be the main culprit leading to adverse physiological sequelae such as RV dilation, dysfunction, and mortality.^23^ More recent studies continue to prove that the PV annulus can be preserved in a selective group of patients with favorable PV anatomy with acceptable residual PS.^10^ However, preservation of PV function without leaving significant PV stenosis continues to be the biggest challenge in patients undergoing pulmonary VS repair. Whether surgical approach has a large impact on clinical outcomes remains controversial as studies such as that of Hickey et al. suggest that surgery type (i.e., VS or TA patch) does not affect long‐term mortality or reintervention.^24^ The important question of “what is an accurate, selective indication for pulmonary VS procedure?” remains. Expanding indication for VS leading has led to increased PS. The latter is strongly associated with the need for reintervention, so the answer to this question grows ever more important. Patients receiving TA patch are typically younger, and this may be related to the difficulty of VS with a smaller valve. With improvements due to early PVR based on MRI and smaller RV incisions by the trans‐RA approach, patients may better tolerate PR. In this case, TAP might be a superior alternative as leaving PR without PS would be preferable to the PS caused by aggressive VS. This may be particularly true of patients with a smaller annulus.

The size of PV annulus has been an important factor for the decision of TA or VS procedures. Awori et al. found that a pulmonary annular *z*‐score less than −1.3 was associated with a 25% chance of gradient >30 mmHg.^25^ The impact of annulus size on postoperative gradients is therefore important to ascertain due to its potential impact on operative mortality and usefulness in deciding between TA patch and VS. Kasturi et al. found that combining either pulmonary annulus index or pulmonary annulus *z*‐scores with main pulmonary artery *z*‐scores were the most accurate methods of predicting the need for TA patch in a large sample of infants undergoing TOF (*n* = 84).^26^ Further supporting the value of *z*‐scores in predicting clinically relevant outcomes post‐TOF, Borodinova et al.'s study of 58 patients undergoing TOF repair found that the RVOT z‐scores less than −3.2 strongly predicted (AUC = 0.979) significant RVOT obstruction post‐surgery.^27^ Stephens et al.'s recent retrospective study of TOF patients found no significant differences in PV annulus *z*‐scores between TA patch patients and those undergoing VS, with *z*‐scores in the latter decreasing from a mean of −2.3 to 1.2 on follow‐up.^28^ Choi et al.'s investigation of morphological and tissue characteristics in determining the surgical approach for 62 TOF patients found the shortcoming of using echocardiographic parameters due to lack of the characterization of RV anatomy and PV. They reported that the sensitivity was 55.0% and the specificity was 77.0% if the *z*‐score cutoff value of PV annulus for TA patch repair was set as −2.0.^29^ These findings suggest that a *z*‐score of −2 provides stable VS outcomes with PV growth and low reintervention. We have expanded PVS as shown in this study. 82% of TOF patients in our study had a VS procedure compared to 46% reported by the STS database.^5^ Additionally, 31 of 61 patients with *z*‐score data (51%) had a value less than −2, achieved by acceptance of RVP less than or equal to 75%. As a result of these factors, we have an increased rate of VS procedure. Yet, we surprisingly found no differences in freedom from reintervention between those with this small annulus and those with larger sizes in our analysis (*p* = .59). This suggests that *z*‐score measured by preoperative echocardiogram is not the best indication for VS versus TA patch, particularly given that echocardiogram measurements underestimate size relative to what is observed in the OR.

We found a strong correlation between intraoperative PG at RVOT measured by TEE and likelihood of having a PV replacement in our VS repair population. The risk of having PV replacement was nearly four times higher when the intraoperative PG was greater than 45 mmHg following a VS repair. We propose that the indication for the conversion to TAP should be greater than 45 mmHg of peak RVOT PG in intraoperative TEE after weaning bypass. A study by Hickey et al. found that right ventricular systolic pressure less than 50 mmHg during repair is associated with a lower less of recurrent stenosis.^30^ This threshold is similar to the value of 45 mmHg identified by our study. Consistent with our findings, Gellis and colleagues found that a residual gradient ≥40 mmHg after balloon dilation was associated with significantly shorter freedom from reintervention—no patients in the no‐reintervention group (*n* = 20) had a residual gradient above this threshold, while 36% of the reintervention group (*n* = 14) did.^31^ Hennein et al. similarly identified residual right ventricular outflow obstruction ≥40 mmHg as a risk factor in their 1995 manuscript.^32^ In contrast, Blais and colleagues recommend tolerating moderate residual stenosis in patients with smaller pulmonary annulus undergoing VS repair, rather than pursuing a TA patch procedure, based on their propensity score‐matched, retrospective analysis of a large TOF patient population between 1980 and 2015.^33^ Thirty‐year survival and reinterventions were both lower in the VS group.

Overzealous pursuit of VS is an area of contention, with several studies suggesting suboptimal outcomes. The acceptance of increased risk of reintervention for outflow track obstruction in exchange for long‐term improvement in RV function is a trade‐off that remains uncertain, particularly as the ostensible benefit remains to be tested.^34^ Lozano‐Balseiro et al.'s study of the midterm results for VS suggests that the increased frequency of the procedure was not without compromise, as over 20% of patients developed significant pulmonary regurgitation.^35^

It has been suggested that intraoperative high RV to LV pressure ratios due to RVOT obstruction may be associated with higher rates of reintervention.^36^ We also found a strong association between high intraoperative RVP and likelihood of having a PV replacement in the future following a VS complete repair. In our study, 87.5% of patients who had RVP greater than or equal to half system pressures had a PV replacement intervention. Our findings suggest that RV pressure should be less than half of systemic pressure, which is supported by Bacha et al.'s determination of cutoff for the RV pressure as 50%.^10^

Furthermore, unlike some studies previously reported,^6^, ^37^ we did not see any change in the PG over time in our study population following initial complete VS procedure. This finding was consistent in both study groups (PV reintervention vs. no‐intervention group), and it underlines the point that there is minimal improvement in PS over time. While Boni et al.'s study showed a decrease in LV/RV ratio at follow‐up, there was no improvement in TEE PG which is consistent with our results. The importance of PG is highlighted by Latus et al.'s findings that a higher RVOT gradient was associated with a composite endpoint of ventricular tachycardia and cardiac death and PG > 25 mmHg predicted adverse cardiovascular events.^38^ When viewed together, these prior studies combine with our results to demonstrate the value of PG as an indicator, as it is both clinically meaningful and not resolved on its own.

### 4.1 Limitations

This is a single‐center retrospective study including a limited number of data points. We thoroughly analyzed all surgical operative and echocardiography reports to obtain pertinent data. Second, our study population is still growing, and long‐term outcomes should be reported in future studies.

## 5 CONCLUSION

In summary, based on our study to reconsider the indication of PVS by reviewing the midterm outcomes of our expanded indication of VS procedure for complete TOF repair, intraoperative decision to convert to transannular patch is warranted if intraoperative postprocedure TEE PG is greater than 45 mmHg or RV pressure is higher than half of the systemic pressure to prevent reintervention.

## CONFLICT OF INTEREST

The authors declare no conflict of interest.

## References

[1] Bacha E . Valve‐sparing options in Tetralogy of Fallot surgery. Semin Thorac Cardiovasc Surg Pediatr Card Surg Annu. 2012;15(1):24‐26. 10.1053/j.pcsu.2012.01.006 22424504

[2] Brown JW , Ruzmetov M , Vijay P , Rodefeld MD , Turrentine MW . Right ventricular outflow tract reconstruction with a polytetrafluoroethylene monocusp valve: a twelve‐year experience. J Thorac Cardiovasc Surg. 2007;133(5):1336‐1343. 10.1016/j.jtcvs.2006.12.045 17467453

[3] Anagnostopoulos P , Azakie A , Natarajan S , Alphonso N , Brook MM , Karl TR . Pulmonary valve cusp augmentation with autologous pericardium may improve early outcome for tetralogy of Fallot. J Thorac Cardiovasc Surg. 2007;133(3):640‐647. 10.1016/j.jtcvs.2006.10.039 17320558

[4] Robinson JD , Rathod RH , Brown DW , et al. The evolving role of intraoperative balloon pulmonary valvuloplasty in valve‐sparing repair of tetralogy of Fallot. Congenit Heart Dis. 2011;142(6):1367‐1373. 10.1016/j.jtcvs.2011.02.047 21703641

[5] Al Habib HF , Jacobs JP , Mavroudis C , et al. Contemporary patterns of management of tetralogy of Fallot: data from the Society of Thoracic Surgeons Database. Ann Thorac Surg. 2010;90(3):813‐820. 10.1016/j.athoracsur.2010.03.110 20732501

[6] Boni L , Garcıa E , Galletti L , et al. Current strategies in tetralogy of Fallot repair: pulmonary valve sparing and evolution of right ventricle/left ventricle pressures ratio. Thorac Surg. 2009;35(5):885‐890. 10.1016/j.ejcts.2009.01.016 19278860

[7] Puranik R , Tsang V , Lurz P , et al. Long‐term importance of right ventricular outflow tract patch function in patients with pulmonary regurgitation. J Thorac Cardiovasc Surg. 2012;143(5):1103‐1107. 10.1016/j.jtcvs.2011.09.039 22056367

[8] Hoashi T , Kagisaki K , Meng Y , et al. Long‐term outcomes after definitive repair for tetralogy of Fallot with preservation of the pulmonary valve annulus. J Thorac Cardiovasc Surg. 2014;148(3):802‐809. 10.1016/j.jtcvs.2014.06.008 24993623

[9] Padalino MA , Pradegan N , Azzolina D , et al. The role of primary surgical repair technique on late outcomes of Tetralogy of Fallot: a multicentre study. Eur J Cardiothorac Surg. 2019;11:ezz270. 10.1093/ejcts/ezz270 31603499

[10] Bacha E . Valve‐sparing or valve reconstruction options in Tetralogy of Fallot surgery. Semin Thorac Cardiovasc Surg Pediatr Card Surg Annual. 2017;20:79‐83. 10.1053/j.pcsu.2016.09.001 28007071

[11] Vida VL , Guariento A , Castaldi B , et al. Evolving strategies for preserving the pulmonary valve during early repair of tetralogy of Fallot: mid‐term results. Congenit Heart Dis. 2014;147:687‐694. 10.1016/j.jtcvs.2013.10.029 24314789

[12] Vida VL , Angelini A , Guariento A , et al. Preserving the pulmonary valve during early repair of tetralogy of Fallot: anatomic substrates and surgical strategies. J Thorac Cardiovasc Surg. 2015;149(5):1358‐1363.e1. 10.1016/j.jtcvs.2015.01.030 25983249

[13] Sasson L , Houri S , Raucher Sternfeld A , et al. Right ventricular outflow tract strategies for repair of tetralogy of Fallot: effect of monocusp valve reconstruction. Eur J Cardiothorac Surg. 2013;43(4):743‐751. 10.1093/ejcts/ezs479 23024233

[14] van der Hulst AE , Hylkema MG , Vliegen HW , et al. Mild residual pulmonary stenosis in tetralogy of Fallot reduces risk of pulmonary valve replacement. Ann Thorac Surg. 2012;94(6):2077‐2082. 10.1016/j.athoracsur.2012.06.065 22981253

[15] Kim YS , Song J , Huh J , Kang IS , Yang JH , Jun TG . The progression of an acceptable pulmonary stenosis immediately after total correction of tetralogy of Fallot. Cardiol Young. 2020;30(6):774‐778. 10.1017/S1047951120000955 32364111

[16] Karl TR . Tetralogy of Fallot: a surgical perspective. Korean J Thorac Cardiovasc Surg. 2012;45(4):213‐224. 10.5090/kjtcs.2012.45.4.213 22880165 PMC3413825

[17] Geva T . Indications for pulmonary valve replacement in repaired tetralogy of Fallot: the quest continues. Circulation. 2013;128(17):1855‐1857. 10.1161/CIRCULATIONAHA.113.005878 24065609 PMC3898939

[18] Knauth AL , Gauvreau K , Powell AJ , et al. Ventricular size and function assessed by cardiac MRI predict major adverse clinical outcomes late after tetralogy of Fallot repair. Heart. 2008;94(2):211‐216. 10.1136/hrt.2006.104745 17135219

[19] Pettersen MD , Du W , Skeens ME , Humes RA . Regression equations for calculation of Z scores of cardiac structures in a large cohort of healthy infants, children, and adolescents: an echocardiographic study. J Am Soc Echocardiogr. 2008;21(8):922‐934. 10.1016/j.echo.2008.02.006 18406572

[20] Cuypers JAAE , Menting ME , Konings EEM , et al. Unnatural history of Tetralogy of Fallot: prospective follow‐up of 40 years after surgical correction. Circulation. 2014;130(22):1944‐1953. 10.1161/CIRCULATIONAHA.114.009454 25341442

[21] Smith CA , McCracken C , Thomas AS , et al. Long‐term outcomes of Tetralogy of Fallot: a study from the Pediatric Cardiac Care Consortium. JAMA Cardiol. 2019;4(1):34. 10.1001/jamacardio.2018.4255 30566184 PMC6439686

[22] Lillehei CW , Varco RL , Cohen M , et al. The first open heart corrections of Tetralogy of Fallot: a 26–31 year follow‐up of 106 patients. Ann Surg. 1986;204(4):490. 10.1097/00000658-198610000-00017 3767482 PMC1251326

[23] Ammash NM , Dearani JA , Burkhart HM , Connolly HM . Pulmonary regurgitation after tetralogy of Fallot repair: clinical features, sequelae, and timing of pulmonary valve replacement. Congenit Heart Dis. 2007;2(6):386‐403. 10.1111/j.1747-0803.2007.00131.x 18377431

[24] Hickey EJ , Veldtman G , Bradley TJ , et al. Late risk of outcomes for adults with repaired tetralogy of Fallot from an inception cohort spanning four decades. Eur J Cardiothorac Surg. 2009;35(1):156‐164. 10.1016/j.ejcts.2008.06.050 18848456

[25] Awori MN , Leong W , Artrip JH , O'Donnell C . Tetralogy of Fallot repair: optimal z‐score use for transannular patch insertion. Eur J Cardiothorac Surg. 2013;43(3):483‐486. 10.1093/ejcts/ezs372 22764146

[26] Kasturi S , Balaji S , Sudhakar A , et al. Accuracy of a new echocardiographic index to predict need for trans‐annular patch in Tetralogy of Fallot. Pediatr Cardiol. 2019;40(1):161‐167. 10.1007/s00246-018-1973-x 30178189

[27] Borodinova O , Mykychak Y , Yemets I . Transesophageal echocardiographic predictor of significant right ventricular outflow tract obstruction after Tetralogy of Fallot repair. Semin Thorac Cardiovasc Surg. 2020;32(2):282‐289. 10.1053/j.semtcvs.2019.09.011 31560970

[28] Stephens EH , Wolfe BL , Talwar AA , et al. Applicability and durability of valve‐sparing Tetralogy of Fallot repair. World J Pediatr Congenit Heart Surgery. 2021;12(5):628‐634. 10.1177/21501351211031242 34597206

[29] Choi SJ , Kwon JE , Roh DE , et al. Importance of pulmonary valve morphology for pulmonary valve preservation in tetralogy of Fallot surgery: comparison of the echocardiographic parameters. Clin Exp Pediatr. 2020;63(5):189‐194. 10.3345/kjp.2019.01060 32024330 PMC7254174

[30] Hickey E , Pham‐Hung E , Halvorsen F , et al. Annulus‐sparing Tetralogy of Fallot Repair: low risk and benefits to right ventricular geometry. Ann Thorac Surg. 2018;106(3):822‐829. 10.1016/j.athoracsur.2017.11.032 29233764

[31] Gellis L , Banka P , Marshall A , Emani S , Porras D . Transcatheter balloon dilation for recurrent right ventricular outflow tract obstruction following valve‐sparing repair of tetralogy of Fallot. Catheter Cardiovasc Interv. 2015;86(4):692‐700. 10.1002/ccd.25930 25914342

[32] Hennein HA , Mosca RS , Urcelay G , Crowley DC , Bove EL . Intermediate results after complete repair of tetralogy of Fallot in neonates. J Thorac Cardiovasc Surg. 1995;109(2):332‐344. 10.1016/S0022-5223(95)70395-0 7531798

[33] Blais S , Marelli A , Vanasse A , et al. Comparison of long‐term outcomes of valve‐sparing and transannular patch procedures for correction of Tetralogy of Fallot. JAMA Netw Open. 2021;4(7):e2118141. 10.1001/jamanetworkopen.2021.18141 34313740 PMC8317016

[34] Emani SM . How to manage the pulmonary valve during repair of Tetralogy of Fallot. Ann Thorac Surg. 2022:S0003‐4975(22)00930‐4. 10.1016/j.athoracsur.2022.06.022 35793715

[35] Lozano‐Balseiro M , Garcia‐Vieites M , Martínez‐Bendayán I , et al. Valve‐sparing Tetralogy of Fallot repair with intraoperative dilation of the pulmonary valve. Mid‐term results. Semin Thorac Cardiovasc Surg. 2019;31(4):828‐834. 10.1053/j.semtcvs.2019.04.007 31005576

[36] Stewart RD , Backer CL , Young L , Mavroudis C . Tetralogy of Fallot: results of a pulmonary valve‐sparing strategy. Ann Thorac Surg. 2005;80(4):1431‐1439. 10.1016/j.athoracsur.2005.04.016 16181883

[37] Nakashima K , Itatani K , Oka N , et al. Pulmonary annulus growth after the modified Blalock‐Taussig Shunt in Tetralogy of Fallot. Ann Thorac Surg. 2014;98(3):934‐940. 10.1016/j.athoracsur.2014.04.083 25038019

[38] Latus H , Stammermann J , Voges I , et al. Impact of right ventricular pressure load after repair of Tetralogy of Fallot. J Am Heart Assoc. 2022;11(7):e022694. 10.1161/JAHA.121.022694 35301850 PMC9075442

